# Hepatocyte-Specific Deletion of Betaine-Homocysteine Methyltransferase Disrupts Methionine Metabolism and Promotes the Spontaneous Development of Hepatic Steatosis

**DOI:** 10.3390/biom16040606

**Published:** 2026-04-20

**Authors:** Ramachandran Rajamanickam, Sathish Kumar Perumal, Ramesh Bellamkonda, Sundararajan Mahalingam, Kurt W. Fisher, Rolen Quadros, Channabasavaiah B. Gurumurthy, Madan Kumar Arumugam, Karuna Rasineni, Kusum K. Kharbanda

**Affiliations:** 1 Research Service, Veterans Affairs Nebraska-Western Iowa Health Care System, Omaha, NE 68105, USA; rrajamanickam@unmc.edu (R.R.); sperumal@unmc.edu (S.K.P.); rbellamkonda@unmc.edu (R.B.); smahalingam@unmc.edu (S.M.); madankumarbio@gmail.com (M.K.A.); karuna.rasineni@unmc.edu (K.R.); 2 Department of Internal Medicine, University of Nebraska Medical Center, Omaha, NE 68198, USA; 3 Department of Biochemistry & Molecular Biology, University of Nebraska Medical Center, Omaha, NE 68198, USA; 4 Department of Pathology, Microbiology and Immunology, University of Nebraska Medical Center, Omaha, NE 68198, USA; kfisher@unmc.edu; 5 Mouse Genome Engineering Core Facility, University of Nebraska Medical Center, Omaha, NE 68198, USA; rolenquadros@gmail.com (R.Q.); cgurumurthy@umc.edu (C.B.G.); 6 Department of Cell and Molecular Biology, University of Mississippi Medical Center, Jackson, MS 39216, USA; 7 Cancer Biology Lab., Centre for Molecular and Nanomedical Sciences, Sathyabama Institute of Science and Technology, Chennai 600119, Tamil Nadu, India

**Keywords:** BHMT, conditional knockout, SAM:SAH ratio, lysosomal dysfunction, liver disease

## Abstract

Betaine-homocysteine methyltransferase (BHMT) is an enzyme involved in one-carbon metabolism and plays a crucial role in maintaining liver health. In this study, we investigated the impact of liver-specific deletion of BHMT on liver dysfunction using a mouse model. We generated BHMT floxed mice and bred them with albumin Cre to generate liver-specific BHMT knockout (BHMT LKO) mice. Liver tissues harvested from six-month-old chow-fed BHMT floxed and LKO mice were characterized through histological, biochemical, and molecular analyses. BHMT LKO mice displayed a complete loss of hepatic expression of BHMT mRNA, protein and enzyme activity. Histopathological analysis revealed the development of hepatic steatosis in BHMT LKO mice compared to the floxed mice. These morphological changes were supported by biochemical analysis showing elevated levels of hepatic triglycerides in conjunction with a profound decrease in the methylation potential (i.e., reduced S-adenosylmethionine (SAM): S-adenosylhomocysteine (SAH) ratio), which was mainly driven by a six- to sevenfold increase in SAH levels. BHMT LKO mice also exhibited increased lipid peroxidation and lysosomal dysfunction compared to floxed mice. Early signs of inflammation were seen in the livers of BHMT LKO mice of both sexes, as evident from significant increase in CD68-positive cells and interleukin 1β levels. Additionally, there was a moderate increase in fibrosis, as evidenced by the upregulated expression of α-smooth muscle actin and collagen II levels and the histological assessment of picrosirius red-stained liver sections of BHMT LKO mice of both sexes compared to their respective counterparts. These findings demonstrate that hepatic BHMT deficiency promotes lipid accumulation, lysosomal/proteasomal dysfunction, and early inflammatory and fibrotic changes in the liver by reducing the methylation potential. Collectively, our results underscore BHMT as a critical regulator of liver homeostasis and a potential therapeutic target in liver-related disorders.

## 1. Introduction

Currently, liver diseases account for nearly 2 million deaths globally each year, resulting in significant economic and social burdens worldwide [1]. The liver plays a central role in maintaining metabolic homeostasis, including processes such as amino acid metabolism, lipid handling, and detoxification [2]. Methionine metabolism is critically important in the liver, where over 85% of the body’s methylation reactions occur, ensuring a continuous supply of methyl groups for essential cellular functions [3]. Within the methionine metabolism pathway, the most critical reaction is the removal of two detrimental metabolites, homocysteine and S-adenosylhomocysteine (SAH), through a folate and vitamin B12-dependent reaction catalyzed by methionine synthase [4]. Betaine-homocysteine methyltransferase (BHMT) is an alternate pathway that contributes up to 50% of homocysteine remethylation in the liver [5,6,7]. Liver BHMT ranks among the most highly expressed hepatic proteins [8,9] that uses betaine to not only remove homocysteine and SAH [10] but also to regenerate S-adenosylmethionine (SAM). This process helps preserve overall methylation potential by maintaining the hepatic SAM:SAH ratio [10,11].

Our laboratory has made seminal contributions in demonstrating that alcohol consumption alters the methionine metabolic pathway in the rodent liver due to impaired methionine synthase activity [11] and decreased betaine levels [12,13]. These ethanol-induced alterations impair both pathways of homocysteine remethylation, causing a reduction in methylation potential through lowering of the SAM:SAH ratio [11]. We have further shown that supplementing betaine in the diet protects against the development of alcohol-associated liver disease (ALD), even if it does not reverse the impairment in the methionine synthase-mediated remethylation pathway [10]. Similarly, betaine deficiency has been observed in patients with metabolic dysfunction-associated liver disease (MASLD), and betaine supplementation has been shown to reverse many hallmark features of this liver disease in both animal models and patients [14,15,16,17,18,19]. Importantly, whole-body BHMT knockout (KO) mice spontaneously develop liver injury despite increased hepatic concentrations of the enzyme’s substrate, betaine [20]. These studies highlight the importance of the BHMT–betaine nexus in maintaining liver homeostasis, as the loss of either can lead to hepatic dysfunction. Additionally, ischemia–reperfusion hepatic injury was shown to be more severe in BHMT KO, whereas BHMT overexpression mitigated these effects [21]. Similarly, knockdown of BHMT in hepatocytes aggravated acetaminophen-induced liver injury [22], while BHMT transgenic mice were resistant to alcohol- or high methionine low folate diet-induced hepatic steatosis [23]. While these studies indicated an important role of BHMT in preventing liver disease of diverse etiologies, research conducted in various laboratories has also shown that this enzyme plays an important role in other organs, as recently reviewed [24,25].

In the last two decades, it has become evident that the cross talk between the gut–liver and adipose–liver systems contributes to the pathogenesis of liver diseases [26,27]. Consequently, many therapeutic approaches to prevent, attenuate, or treat chronic liver diseases are aimed at modulating adipose dysfunction and gut dysbiosis and improving intestinal barrier function. One such effective method is use of betaine, a choline-derived metabolite and a methyl group donor with antioxidant, anti-inflammatory, and osmoprotectant properties. Moreover, its ability to prevent adipose dysfunction, regulate protective gut microbiota, and maintain intestinal barrier integrity makes it a promising therapeutic agent for preventing the development of chronic liver diseases, including ALD and MASLD [16,18,25,28,29,30]. However, the relative contrition of BHMT in each of these organs (liver, adipose, gut) in preventing liver disease remains unknown and needs clarification. Based on these considerations, the current study aimed to assess the molecular mechanisms underlying the effect of organ-specific BHMT loss on metabolic functions in the liver. To define the crucial role of BHMT in maintaining liver function, we generated BHMT floxed and liver-specific BHMT KO (LKO) mice. In this study, we performed phenotypic characterization of the newly generated mouse model using histological, biochemical, and molecular assays. Our data provide strong evidence that BHMT is essential for hepatic metabolic processes related to methylation-dependent pathways. We used both male and female mice to also investigate the potential sex-specific roles of hepatic BHMT, which also remains poorly understood.

## 2. Materials and Methods

### 2.1. Generation of BHMT Floxed and LKO Mice

**Figure 1:**
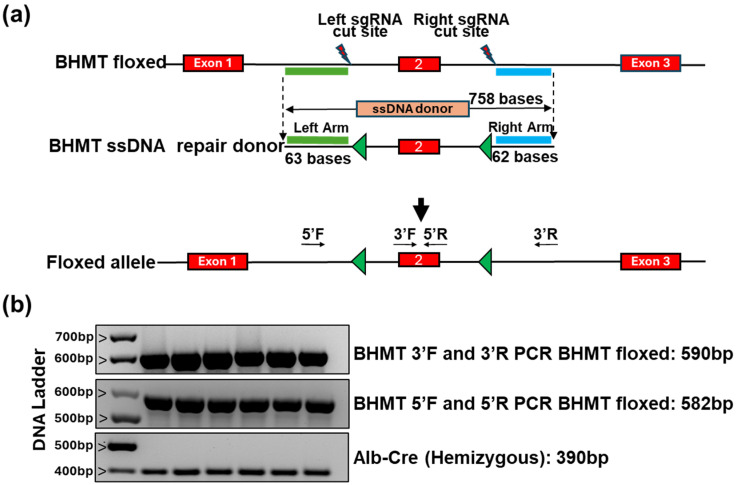
(**a**) Schematic showing the wild-type locus and the floxing design for targeting exon 2 of the betaine-homocysteine methyltransferase (*BHMT)* gene. The floxed allele was generated using the Easi-CRISPR method [31,32], which uses long single-stranded DNA as the repair donor template. The lengths of ssDNA, homology arms, and the distance between the two LoxP sites are shown. (**b**) Genotyping of the BHMT floxed mice using PCRs, one each for the two LoxP sites (5′ LoxP PCR, 5′ forward (5′F) + 5′ reverse (5′R) primers; 3′ LoxP PCR, 3′F (3′F) + 3′R (3′R) primers) and hepatocyte-specific BHMT-knockout mice. The expected sizes of BHMT floxed and albumin (Alb)–Cre PCR amplicons are indicated.

The Easi-CRISPR method [31,32] was used to generate the model by floxing exon 2 (ENSMUSE00001280457) of BHMT. The LoxP sites were inserted at 194 and 216 bases upstream and downstream of the exon, respectively, using two guide RNA sequences (ATACATGCCAAGATTGGCTC and CTAGACTCATCCAAAGTGAA). Long single-stranded DNA containing the flanking LoxP sites and 63-base and 72-base homology arms was used as a donor in the Easi-CRISPR method [31,32]. Mouse transgenesis experiments to generate zygotes, microinjection, and their transfer to pseudopregnant females were performed as described in [33]. The C57BL6/J strain was used for generating the model. Primers used for genotyping the offspring were as follows. For 5′ LoxP; BHMT 5F: GCTCAGGAGCCAAATTCCATGTTC, BHMT 5R: GTTTAGAATGGAGCGTGTACCA. For 3′ LoxP: BHMT 3F: GAAACTCCTTTCTCTCCCTCATC and BHMT 3R: CAAATTCTAGGGCAGGTAGCT. A schematic of the floxed allele and the genotyping of the floxed mice is shown in Figure 1. Upon breeding the floxed allele to a Cre line, the transcript was expected to produce only truncated protein of about 11 amino acids (from exon 1), the spliced distal exons (if this occurs) to produce out-of-frame protein, and the transcript to undergo nonsense-mediated decay. Albumin Cre mice (JAX stock number 003574) were used for breeding with the BHMT floxed mouse to generate liver-specific knockout of the BHMT line (BHMT LKO).

### 2.2. Animals

C57BL/6 BHMT floxed and LKO mice were housed at the Omaha Veterans Affairs Medical Center Veterinary Medical Unit, accredited by the American Association for Accreditation of Laboratory Animal Care. These mice were on a 12 h:12 h light/dark cycle at an ambient temperature (20–25 °C) and 40%–60% relative humidity, with free access to standard-chow-diet food and water. The study protocol was approved by the Institutional Animal Care and Use Committee (IACUC) of Omaha Veterans Affairs Medical Center (IRBnet 1583585 and IRBnet 1674572, approved 14 May 2020 and 29 August 2023, respectively). Genotypes were confirmed by PCR of tail DNA using primers for floxed BHMT alleles and Cre transgene. Knockout efficiency was validated by Western blotting of liver protein extracts, probed with anti-BHMT and β-actin antibodies to confirm the absence of BHMT in LKO mice. Six-month-old BHMT floxed mice and those with confirmed BHMT deletion were used for experimental analyses.

The animals were anesthetized with isoflurane (5% to effect), and blood and liver samples were collected and appropriately processed for further analysis. The chest was opened, and death was induced through a combination of exsanguination and pneumothorax. These methods are consistent with the recommendations of the Panel on Euthanasia of the American Veterinary Medical Association (*AVMA Guidelines for the Euthanasia of Animals*: 2020 Edition, AVMA, Schaumburg, IL, USA).

### 2.3. Histopathological Examination

A portion of the liver harvested from the experimental animals were fixed immediately in formalin for 48 h before being transferred to 75% ethanol. Liver tissue was then processed by the UNMC Tissue Sciences Facility, paraffin-embedded, sectioned at 5 μm, and stained with hematoxylin and eosin (H&E) for assessing morphology or with picrosirius red for examining collagen deposition. These evaluations were conducted by a board-certified pathologist (K.W.F.), and the images were captured at various magnifications with a Keyence BZ-810 microscope (Itasca, IL, USA).

### 2.4. Serum Markers of Liver Injury

Serum levels of alanine aminotransferase (ALT) were measured at the clinical laboratory of the VA Nebraska Western Iowa Health Care System.

### 2.5. Analysis of Liver BHMT Activity and SAM and SAH Levels

BHMT enzyme activity in the liver lysates was measured following the method of Ericson and Harper [34]. Additionally, liver lysates prepared in 0.5 N perchloric acid were subjected to high-performance liquid chromatography analysis to determine SAM and SAH levels to calculate the SAM:SAH ratio (aka methylation potential), as detailed in previous methods [11].

### 2.6. Triglycerides and Non-Esterified Fatty Acid (NEFA) Levels

Triglyceride levels in serum and liver lipid extracts [35] were quantified using a diagnostics kit (TR22421, Thermo Fisher Scientific, Waltham, MA, USA) according to the manufacturer’s instructions, as detailed previously [11]. Serum NEFA levels were determined using a kit from Fujifilm, Lexington, MA, USA.

### 2.7. Hepatic Reactive Oxygen Species (ROS) and Lipid Peroxidation

Liver ROS were measured using 2′7′-dichlorodihydrofluorescein diacetate, as detailed [36]. Formation of the oxidized fluorescent derivative dichlorofluorescein was monitored at 485 nm (excitation) and 530 nm (emission). Data are expressed as fluorescence units and are normalized for protein concentration, measured by the Bradford dye-binding assay [37]. We determined hepatic lipid peroxidation by measuring thiobarbituric acid-reactive substances (TBARS) as detailed in [38] using purified malondialdehyde (MDA) as the standard.

### 2.8. Lysosomal and Proteasomal Enzyme Activity

Liver lysosomal enzyme activity, including lysosomal acid lipase (LAL), cathepsin B, and cathepsin L, was measured as previously described [39]. Additionally, the chymotrypsin-like activity of the 20S and 26S proteasomes was measured using an SUC-LLVY-AMC fluorogenic substrate (G1100, UBPBio Inc., Dallas, TX, USA) following the protocol outlined in our previous publication [39]. Measurements were taken at excitation and emission wavelengths of 380 and 460 nm, respectively, using a fluorimeter (Spectramax M5, Molecular Devices, San Jose, CA, USA). Furthermore, the trypsin-like activity of the 20S proteasome in liver tissue was assessed using a UBPBio diagnostic kit (G3100), as described in our earlier publication [39].

### 2.9. Gene Expression

RNA was isolated with a PureLink™ RNA minikit (12183018A, Invitrogen, Waltham, MA, USA) following the manufacturer’s instructions. RNA was quantified spectrophotometry (NanoDrop Technologies, Wilmington, DE, USA), and 200 ng of RNA was reverse-transcribed to cDNA using a high-capacity reverse-transcription kit (4368813, Applied Biosystems, Waltham, MA, USA). We quantified the relative levels of mRNAs encoding BHMT, cluster of differentiation 36 (CD36) and cell death-inducing DFFA-like effector C (CIDEC) using TaqMan Universal Master Mix II (4440038, Applied Biosystems, Waltham, MA, USA) with fluorescence-labeled FAM primers (TaqMan gene expression systems, 4331182, Applied Biosystems, Waltham, MA, USA) using a 7500 real-time PCR detection system (Applied Biosystems, Waltham, MA, USA). The relative quantity of each RNA transcript was calculated by its threshold cycle (Ct) after subtracting that of the reference cDNA (β-actin). Data are expressed as the relative quantity (RQ) of each transcript. The primer details are listed in Supplementary Table S1.

### 2.10. Protein Expression

Liver tissues were lysed in RIPA buffer and total protein concentration determined in the supernatants with the BCA protein assay method. For Western blot analysis, samples were separated by sodium dodecyl sulfate–polyacrylamide gel electrophoresis and transferred to 0.45 µm nitrocellulose membranes (Bio-Rad Laboratories, Hercules, CA, USA) following standard protocols. Samples were analyzed using commercial antibodies, described in Supplemental Table S2. We visualized the proteins using ECL substrate (170-15060, Bio-Rad Laboratories, Hercules, CA, USA) on a Chemidoc MP imaging system (Bio-Rad Laboratories, Hercules, CA, USA). The intensities of immunoreactive protein bands were quantified using ImageJ (version 1.54g). The complete set of original Western blot images is provided in Supplementary File S1.

To validate the BHMT LKO mouse model, we also assessed BHMT expression in non-liver lysates by Western blot analysis. 

### 2.11. Immunofluorescence Staining

Immunofluorescence staining for cluster of differentiation 68 (CD68) was performed as described previously [40]. Briefly, paraffin-embedded liver sections were deparaffinized in xylene and rehydrated in ethanol. Following deparaffinization, slides were subjected to antigen retrieval with 10 mmol/L sodium citrate buffer (pH 6) at 95 °C for 15 min. Blocking was accomplished by incubating in Super Block (AA999, ScyTek Laboratories Inc., West Logan, UT, USA) for 1 h at room temperature. Sections were incubated overnight with an antibody against CD68 at 4 °C. The sections were then thoroughly washed with PBS followed by incubation with an ImmPRESS^®^-AP horse anti-rabbit IgG polymer reagent, alkaline phosphatase (MP-5401, Vector Laboratories Inc., Newark, CA, USA), and exposure to a Vector Red substrate kit and alkaline phosphatase (SK-5100, Vector Laboratories, Newark, CA, USA) for signal development. Nuclei counterstaining was conducted using 1 µg/mL DAPI for 1 min, and the slides were imaged under a Keyence BZ-X810 microscope (Itasca, IL, USA). The antibodies used in this study are listed in Supplementary Table S2.

### 2.12. Statistical Analysis

The experimental data were analyzed using MS Excel and GraphPad Prism (version 10.5.0). The results are presented as means ± SEM. Each data point on the bar charts in the figures is represented by a sex symbol to indicate whether the data pertain to male (♂) or female (♀) mice. Statistical significance between the two groups was determined using Student’s *t*-test, and a *p*-value of less than 0.05 was considered statistically significant.

## 3. Results

### 3.1. BHMT LKO Phenotype

**Figure 2:**
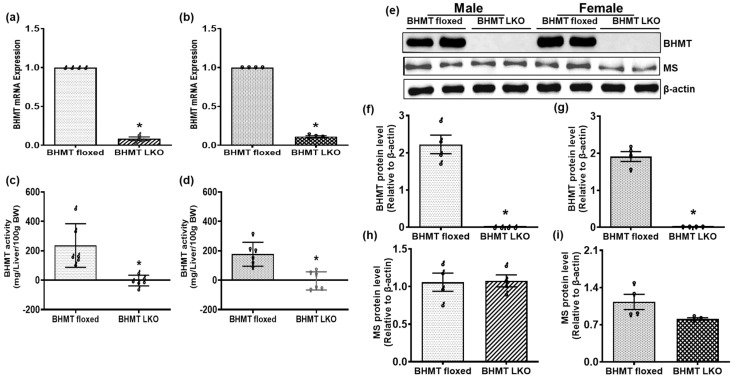
Validation of liver-specific betaine-homocysteine methyltransferase (BHMT) knockout (LKO) mice. Hepatic BHMT mRNA expression and enzymatic activity in six-month-old (**a**,**c**) male (♂) and (**b**,**d**) female (♀) chow-fed floxed and LKO mice. (**e**) Western blot showing BHMT and methionine synthase (MS) protein expression in representative livers and densitometric quantification using ImageJ (version 1.54g) of (**f**,**g**) BHMT and (**h**,**i**) MS in (**f**,**h**) male and (**g**,**i**) female floxed and LKO mice. Data are presented as means ± SEM (n = 6); * *p* < 0.05.

Minimal expression of liver BHMT mRNA and activity was observed in both male and female LKO mice (Figure 2a–d), unlike the floxed control mice. This was confirmed by Western blot analyses, which revealed a complete loss in BHMT protein expression in livers of LKO mice, while there were no significant differences in methionine synthase protein levels between the floxed and LKO mice (Figure 2e–i). Similar BHMT expression was also observed in a non-liver tissue (kidney) from both BHMT floxed and LKO mice (Figure S1), further supporting the validity of the BHMT LKO mouse model.

### 3.2. Hepatocyte-Specific Deletion of BHMT Promotes the Spontaneous Development of Liver Steatosis Compared to Age-Matched BHMT Floxed Mice

**Figure 3:**
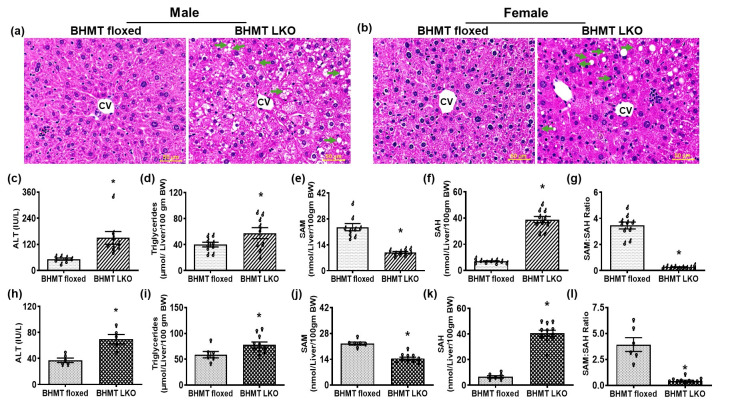
Spontaneous development of hepatic steatosis in association with lower methylation potential in six-month-old chow-fed liver-specific betaine-homocysteine methyltransferase (BHMT) knockout (LKO) mice. Representative photomicrographs of H&E-stained liver sections (scale—50 µm) of (**a**) male and (**b**) female LKO mice depicting numerous large lipid droplets (green arrows). The central vein is marked CV. Serum ALT and levels of hepatic triglycerides, S-adenosylmethionine (SAM), S-adenosylhomocysteine (SAH), and SAM:SAH ratio in (**c**–**g**) male (♂) and (**h**–**l**) female (♀) BHMT floxed and LKO mice. Data are presented as means ± SEM (n = 6); * *p* < 0.05.

Following euthanasia, we observed no significant differences in body or adipose tissue weight between LKO mice and sex-matched floxed mice (Figure S2a,b,e,f). However, significantly increased liver weight was noted in both male and female LKO mice (Figure S2c,g). Consistently, the liver-to-body weight ratio was also significantly increased in BHMT LKO mice, indicating that the loss of BHMT alters liver composition (Figure S2d,h). To further confirm the underlying pathophysiological changes in BHMT LKO mice, we conducted histological assessment of H&E-stained liver sections, which revealed accumulation of lipid droplets in both male and female LKO mice compared to floxed mice (Figure 3a,b). This was accompanied by significant increases in serum ALT levels in LKO mice of both sexes compared to their floxed counterparts (Figure 3c,h). In agreement with the histological examinations, biochemical quantification revealed a significantly elevated level of triglycerides in livers of both male and female LKO mice fed a chow diet (Figure 3d,i). The observed hepatic lipid accumulation led us to examine whether BHMT LKO mice exhibited alterations in hepatic SAM and SAH levels and their ratio, critical elements for maintaining methylation reactions and preventing lipid accumulation and progressive liver injury. We observed a 33–50% decrease in hepatic SAM levels, while there was a five- to sevenfold increase in SAH levels and hence a profound decline in the hepatocellular SAM:SAH ratio in LKO mice of both sexes compared with their counterpart floxed controls (Figure 3e–g,j–l).

**Figure 4:**
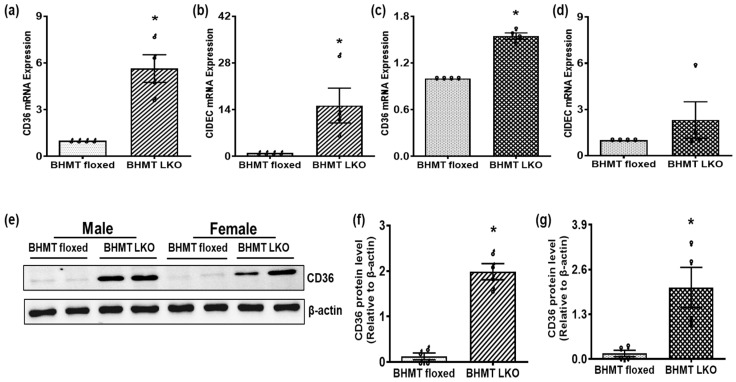
Alterations in lipid-handling pathways in six-month-old chow-fed liver-specific betaine-homocysteine methyltransferase (BHMT) knockout (LKO) mice. (**a**,**c**) Hepatic cluster of differentiation 36 (CD36) and (**b**,**d**) cell death-inducing DFFA-like effector (CIDEC) mRNA expression in (**a**,**b**) male (♂) and (**c**,**d**) female (♀) BHMT floxed and LKO mice. (**e**) Western blotting showing CD36 protein expression in representative livers and its quantification using ImageJ in (**f**) male and (**g**) female BHMT floxed and LKO mice. Data are presented as means ± SEM (n = 6); * *p* < 0.05.

Elevated hepatic triglyceride levels were correlated with altered expressions of CD36 and CIDEC, key regulators in the development of steatosis. To evaluate changes in lipid-handling pathways, we examined CD36, which regulates fatty acid uptake and intracellular transport. BHMT LKO mice of both sexes showed significantly increased hepatic expression of mRNA encoding for CD36 (Figure 4a,c), while that for CIDEC was significantly increased in male (Figure 4b), but unchanged in female (Figure 4d) mice compared to their respective floxed mice. Western blot analysis confirmed the upregulation of CD36 protein in livers of both male and female BHMT LKO mice compared to floxed mice (Figure 4e–g). We also measured serum triglyceride and non-esterified fatty acid (NEFA) levels and found no differences between male or female BHMT LKO mice and their sex-matched counterparts (Figure S3a–d).

### 3.3. Hepatocyte-Specific Deletion of BHMT Promotes Liver Dysfunction

**Figure 5:**
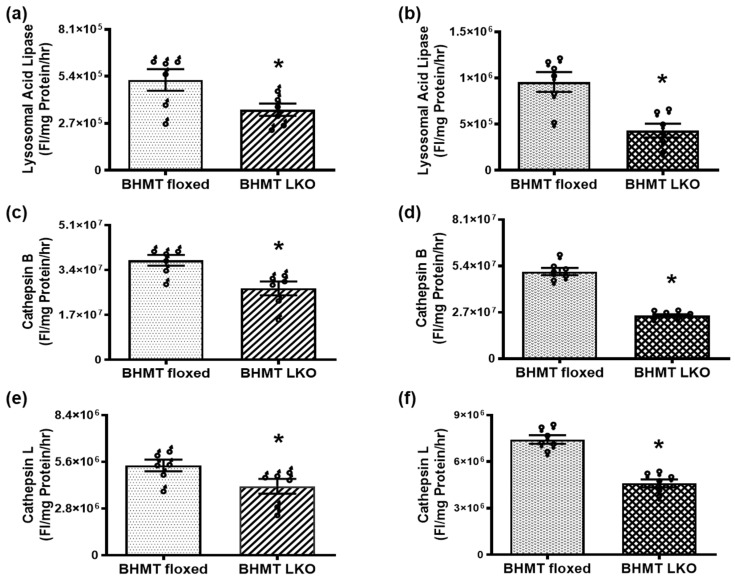
Reduction in lysosomal enzyme activity in six-month-old chow-fed liver-specific betaine-homocysteine methyltransferase (BHMT) knockout (LKO) mice. (**a**,**b**) Lysosmal acid lipase, (**c**,**d**) cathepsin B, and (**e**,**f**) cathepsin L activity in the livers of (**a**,**c**,**e**) male (♂) and (**b**,**d**,**f**) female (♀) BHMT floxed and LKO mice. Data are presented as means ± SEM (n = 6); * *p* < 0.05.

To further understand the mechanisms of BHMT LKO affecting liver function, we measured the enzymatic activity of a key lysosomal enzyme involved in lipid catabolism, namely lysosomal acid lipase (LAL). As illustrated, hepatic LAL activity declined significantly in BHMT LKO male (Figure 5a) and female (Figure 5b) mice compared to floxed mice. Cathepsins are lysosomal proteases that are integral in protein turnover and cellular metabolism. Like LAL, the activity of cathepsins B and L showed a clear trend towards significant decreases in LKO mice of both sexes (Figure 5c–f), indicating impaired lysosomal lipid degradation and protein turnover in these mice compared to the floxed mice.

### 3.4. Mice with Hepatocyte-Specific Deletion of BHMT Exhibit Higher Oxidative Stress Compared to BHMT Floxed Mice

**Figure 6:**
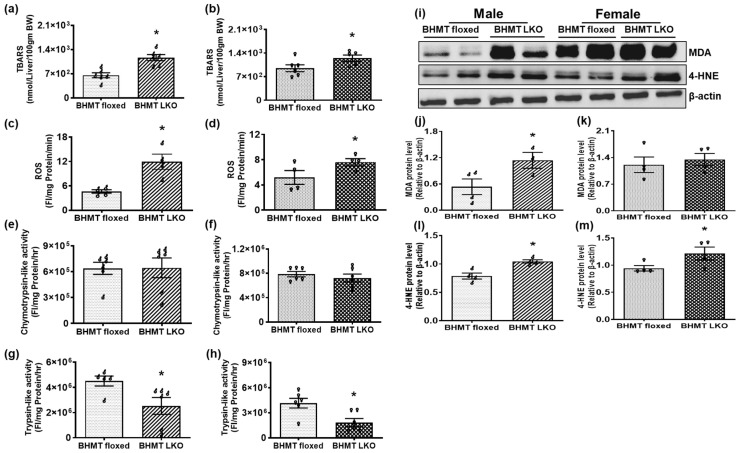
Liver-specific deletion of betaine-homocysteine methyltransferase (BHMT) causes oxidative stress in six-month-old chow-fed mice. (**a**,**b**) Thiobarbituric acid reactive substances (TBARS), (**c**,**d**) reactive oxygen species (ROS), (**e**,**f**) and chymotrypsin- and (**g**,**h**) trypsin-like proteasome activity in livers of (**a**,**c**,**e**,**g**) male and (**b**,**d**,**f**,**h**) female BHMT floxed and LKO mice. (**i**) Western blot showing oxidative stress markers, malondialdehyde (MDA)–protein and 4-hydroxynonenal (4-HNE)–protein adducts in representative livers and their densitometric quantification using ImageJ in (**j**,**l**) male (♂) and (**k**,**m**) female (♀) BHMT floxed and LKO mice. Data are presented as means ± SEM (n = 4–6); * *p* < 0.05.

We anticipated that hepatic steatosis and impaired lysosomal dysfunction would promote oxidative stress, and therefore measured ROS and lipid peroxidation products. Hepatic TBARS levels were significantly elevated in BHMT LKO mice compared with floxed controls, with consistent increases observed in both male (Figure 6a) and female (Figure 6b) mice, indicating heightened lipid peroxidation. Similarly, hepatic ROS levels were significantly elevated in BHMT LKO mice of both sexes (Figure 6c,d), suggesting increased susceptibility of oxidative damage to cellular proteins, DNA, and lipids. Additionally, Western blot analysis supported these findings. We observed significant increases in malondialdehyde (MDA)-protein adducts in males, whereas females displayed no detectable changes (Figure 6i–k). In contrast, 4-hydroxynonenal (4-HNE)–protein adducts were increased in BHMT LKO livers of both sexes, confirming elevated oxidative damage (Figure 6i,l,m). We further assessed proteasomal function by measuring liver chymotrypsin-like and trypsin-like activity. While we observed no change in hepatic chymotrypsin-like activity between the LKO and floxed mice of either sex (Figure 6e,f), significant decreases in trypsin-like activity were seen in both male and female LKO mice compared with their floxed counterparts (Figure 6g,h).

### 3.5. Hepatocyte-Specific Deletion of BHMT Promotes Early Inflammatory and Fibrotic Changes in the Liver

**Figure 7:**
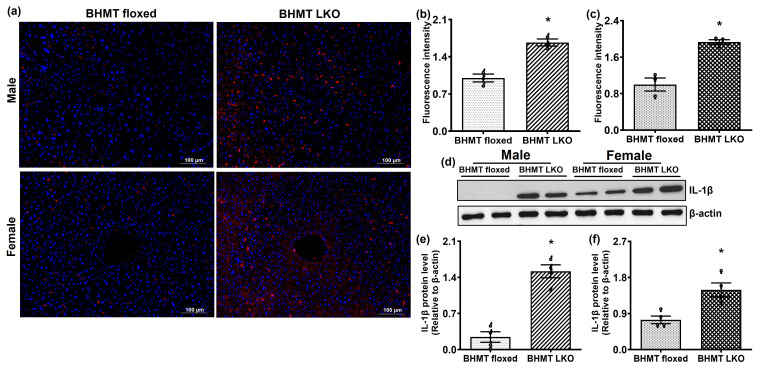
Inflammatory changes in six-month-old chow-fed liver-specific betaine-homocysteine methyltransferase (BHMT) knockout (LKO) mice. (**a**) Fluorescent images (scale—100 µm) of liver sections of representative BHMT floxed and LKO male and female mice immunostained with CD68 (red) and DAPI (blue). Fluorescence intensity of CD68 in livers of (**b**) males (♂) and (**c**) females (♀) relative to BHMT floxed mice. (**d**) Western blot showing interleukin 1β (IL-1β) expression in representative livers and its quantification using ImageJ in (**e**) male and (**f**) female BHMT floxed and LKO mice. Values are means ± SEM of 4 randomly selected fields from each section and data pooled from sections obtained from individual animals (n = 4); * *p* < 0.05.

Having demonstrated that BHMT LKO mice exhibited indices in liver injury and altered lipid metabolism, we further investigated whether BHMT deficiency promotes inflammatory changes. We performed immunofluorescence staining of liver sections of BHMT floxed and LKO mice to assess macrophage infiltration. We observed an increased number of CD68-positive cells in livers of BHMT LKO compared to floxed controls, indicating that loss of BHMT promotes hepatic macrophage accumulation (Figure 7a). Quantitative analysis confirmed a significant increase in CD68-positive cells in LKO mice of both sexes (Figure 7b,c). To further characterize the inflammatory response, we determined gene and protein expression of interleukin 1β (IL-1β) expression, which confirmed evidence of early inflammation in LKO mice (Figure 7d–f).

**Figure 8:**
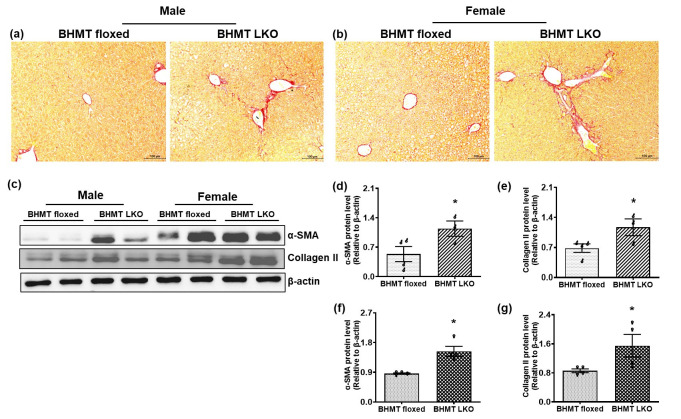
Six-month-old chow-fed liver-specific betaine-homocysteine methyltransferases (BHMT) knockout (LKO) mice exhibit moderate early fibrotic changes. (**a**,**b**) Representative images of picrosirius red-stained sections (scale—100 µm) showing collagen deposition in livers of male and female BHMT floxed and LKO mice. (**c**) Western blotting showing markers related to fibrosis (α-smooth muscle action (α-SMA) and collagen II) in representative livers and their densitometric quantification using ImageJ in (**d**,**e**) male (♂) and (**f**,**g**) female (♀) BHMT floxed and LKO mice. Data are presented as-means ± SEM (n = 6); * *p* < 0.05 versus floxed mice.

Picrosirius red staining confirmed moderate increases in collagen deposition in LKO mice compared to floxed controls, indicating the presence of early fibrogenesis (Figure 8a,b). Supporting this observation, Western blot analysis showed moderate increases in levels of α-SMA and collagen protein in BHMT LKO livers (Figure 8c–g), consistent with hepatic stellate cell activation and disease progression toward liver fibrosis.

## 4. Discussion

Previous studies in mouse models have focused primarily on specific metabolic alterations resulting from global BHMT KO in mouse models [20,41,42]. To our knowledge, the present study is the first to provide an analysis of a hepatocyte-specific BHMT-deficient mouse model. We generated BHMT floxed mice and hepatocyte-specific knockout mice to determine the phenotypic profile and impact on hepatic liver injury development in these mice. The main findings are summarized as follows. (1) Hepatocyte-specific deletion of BHMT resulted in a complete loss of BHMT mRNA and protein expression in the liver, which was confirmed by enzyme activity measurement. (2) Under standard-chow-diet conditions with normal access to food and water, BHMT LKO mice of both sexes at a relatively young age of 6 months exhibited histological alterations characteristic of increased hepatic triglyceride levels. (3) The BHMT LKO mice exhibited lower methylation potential, as evidenced by a decreased SAM:SAH ratio. (4) Impairment in lysosomal hydrolase activity was observed in both male and female BHMT LKO mice. (5) Inhibition in trypsin-like proteasome correlated with the increased oxidative stress seen in the livers of the BHMT LKO mice. (6) Hepatic steatosis was accompanied by early inflammatory and fibrotic changes in the livers of BHMT LKO mice. These findings highlight a critical role for BHMT in maintaining hepatic methylation potential, which regulates lipid homeostasis, oxidative stress and lysosomal/proteasomal function, and in preventing the spontaneous development of liver injury. The observed decreases in hepatocellular SAM:SAH ratios in BHMT LKO mice suggest that the loss of BHMT, despite an intact and normal alternate MS-catalyzed pathway of remethylation, impairs a wide array of methylation-dependent cellular processes. This aligns with previous reports implicating BHMT in metabolic regulation and disease susceptibility [9,43,44,45]. Moreover, the progressive advancement of liver injury beyond steatosis, while moderate in intensity, at 6 months of age underscores the pathological relevance of BHMT deficiency in advanced liver disease development [20].

Previous studies have linked reduced BHMT expression and genetic variants with increased risk of liver disorders and other metabolic diseases, but information underlying the metabolic mechanisms remain obscure [20,46]. Since BHMT is expressed in several other important organs, including adipose, intestine, kidneys, and pancreas, and all these organs communicate with the liver, studies in whole-body BHMT KOs cannot fully appreciate which of these organs could be adversely affecting the liver. Previous studies in whole-body BHMT KOs revealed fatty liver formation in 5-week-old mice, with more than a sixfold increase in hepatic triglycerides [20]. Our study with LKO mice showed only a ~1.5-fold increase in hepatic triglycerides at 6 months of age and is better designed to provide novel insights into how liver-specific BHMT deficiency disrupts hepatic lipid handling, impairs organelle function, and alters liver adaptive responses. We observed a marked increase in liver weight and serum ALT levels in 6-month-old BHMT LKO mice of both sexes. However, there was no significant change in body or adipose tissue weight in BHMT LKO mice compared to BHMT floxed mice. In contrast, studies conducted in whole-body BHMT KOs revealed decreased body weight, fat mass, and adipose tissue compared to wild-type mice [41,42], indicating that adipose lipolysis in whole-body BHMT KOs may be contributing to the sixfold increased hepatic triglycerides seen in these mice [20].

Histological analysis of liver tissue from BHMT LKO mice fed a standard chow diet revealed the presence of lipid droplets, consistent with the biochemical evidence of a significant accumulation of hepatic triglycerides. This lipid accumulation likely reflects a multifactorial metabolic disturbance resulting from a loss of methylation potential due to liver BHMT deficiency.

Particularly relevant to this study are several publications from our and other laboratories showing that a reduction in the hepatocellular SAM:SAH ratio affects several critical pathways related to lipid metabolism by directly promoting lipogenesis while impairing the synthesis and secretion of very-low-density lipoprotein synthesis and export [47,48,49,50]. Furthermore, reduced methylation potential can influence the transcriptional regulation of other proteins related to lipid metabolism [49]. One such protein is CD36, a fatty acid translocase, which was significantly upregulated at both the mRNA and protein levels in BHMT LKO livers in both sexes compared to their floxed counterparts. Increased CD36 expression is known to promote hepatic fatty acid influx and has been implicated in the development of steatosis and progression to fibrosis [51]. CIDEC is a lipid droplet-associated protein that plays a critical role in the development of hepatic steatosis, since its overexpression promotes triglyceride accumulation in both adipocytes and hepatocytes [52]. The present study reveals that CIDEC mRNA expression was upregulated in BHMT LKO mice, consistent with the increased hepatic triglyceride levels and histological evidence of lipid droplet accumulation. Together, these findings suggest that BHMT plays a regulatory role in hepatic lipid metabolism through its influence on methylation status and downstream effects on lipid storage pathways.

There appeared to be sex-specific differences in hepatic lipid deposition, as both microsteatosis (smaller lipid droplets) and macrosteatosis (larger lipid droplets) were observed in the livers of male BHMT LKO mice, whereas only macrosteatosis was observed in their female counterparts. These differences likely stem from altered lipid droplet physiology in male versus female BHMT LKO mice. Understanding these differences in lipid droplet dynamics between male and female 6-month-old liver-specific BHMT LKO mice will be the focus of our future studies.

Growing evidence implies an association between lysosomal dysfunction and liver-related pathologies [53]. Our previous studies implicated a direct effect of the ethanol-induced loss of methylation potential on lysosomal function and its restoration by betaine treatment that normalized the SAM:SAH ratio [39]. Thus, we examined the liver lysosomal activity and found that the activity of key lysosomal enzymes cathepsin B, cathepsin L, and LAL were significantly reduced in BHMT LKO mice. These enzymes play essential roles in lysosomal function and lipid degradation, particularly through lipophagy, the autophagic breakdown of lipid droplets [54]. LAL is directly responsible for hydrolyzing triglycerides and cholesteryl esters, while cathepsins B and L are proteases involved in lysosomal proteolysis and autophagic flux [55,56]. The downregulation of LAL suggests a compromised lysosomal capacity to degrade and recycle lipid droplets, thereby contributing to hepatic triglyceride accumulation [57]. Our results are consistent with a study highlighting that the lysosomal dysfunction seen in BHMT LKO mice is likely to act synergistically with altered lipid metabolism to drive the development of hepatic steatosis and liver disease progression in these mice [20]. Recent reports show that mice with hepatocyte-specific knockdown of LAL exhibit drastic proteome alterations, including dysregulation of multiple proteins related to metabolism, inflammation, liver fibrosis, and cancer [57]. We believe that sustained defective lysosomal degradation could promote inflammation, fibrosis, and hepatocellular stress, providing a mechanistic link between BHMT deficiency and the development of more advanced liver injury as these mice age further.

Overexpression of CIDEC has been shown to increase mitochondrial ROS generation in hepatocytes [58]. In the current study, significant increases in markers of oxidative stress, including elevated levels of ROS and lipid peroxidation products, were observed in the livers of BHMT LKO mice. Consistent with this observation, protein adducts with both lipid peroxidation byproducts, MDA and 4-HNE, were markedly increased. These findings demonstrate that BHMT deficiency in the liver correlates with the degree of lipid accumulation, impaired methylation, and oxidative stress [20,46,59]. However, this increased oxidative stress was unable to mount any hepatic antioxidant response, assessed by determining the expression of catalase and superoxide dismutase 1. While both these enzymes were expressed, their levels did not appear to be sufficiently upregulated in livers of BHMT LKO mice to defend against the heightened oxidative burden. This inadequate antioxidant response may contribute to the spontaneous development of liver injury in BHMT LKO mice.

The reduced trypsin-like activity observed in LKO mice impairs the degradation of damaged or oxidized proteins that accumulate under oxidative stress, which in turn contributes to the severity of liver disease [59]. To support this notion, previous research indeed demonstrated that proteasome activity is impaired by reduced SAM:SAH ratios and is also differentially regulated by oxidative stress, i.e., low oxidative stress impairs proteasome activity, while high oxidative stress does the opposite [60,61]. Interestingly, a recent study from our laboratory on aging promoting spontaneous liver injury development demonstrated similar changes in cathepsin, LAL, and proteasomal activity between sexes in 20- to 22-month-old mice, as seen in the present study on 6-month-old BHMT LKO mice [62]. These observations lend support to the notion that liver-specific BHMT deficiency may be promoting premature aging of the liver.

The alterations in proteasomal activity in addition to perturbing protein homeostasis could also promote/signal the early onset of inflammation and fibrogenesis [63]. To further explore the progression of liver injury beyond steatosis, we assessed macrophage infiltration and confirmed an increased number of CD68-positive cells in liver sections of BHMT LKO mice compared to floxed controls, indicating that loss of BHMT alters hepatic macrophage accumulation. This increased macrophage infiltration was associated with increased IL-1β expression, and densitometric quantification confirmed the relative increase in IL-1β protein abundance in both male and female LKO mice. These inflammatory markers provide evidence of inflammatory responses associated with BHMT LKO mice of both sexes.

We also observed noticeable fibrotic changes in livers of BHMT LKO mice. Picrosirius red staining of liver sections revealed moderately increased collagen deposition in the livers of BHMT LKO mice, indicating early signs of fibrosis development. This was further supported by elevated protein expression of α-smooth muscle actin (α-SMA), a well-established marker of hepatic stellate cell activation [64,65], and increased collagen levels assessed by Western blotting. Together, these findings indicate that the chronic metabolic and oxidative stress induced by BHMT deficiency is sufficient to trigger early fibrotic remodeling of the liver. Together, these data suggest that BHMT deficiency results in a multifaceted pathogenic program that is initiated by reduced methylation potential. This loss of methylation potential leads to impaired lipid handling, oxidative and proteotoxic stress, lysosomal/proteasomal dysfunction, and an early inflammatory/fibrotic response. This comprehensive profile highlights the essential role of hepatic BHMT in maintaining methylation potential and provides mechanistic insights into how its dysregulation may contribute to liver disease progression. Ongoing and future studies are investigating whether more severe liver injury occurs in BHMT LKO mice with advancing age and therapeutic strategies targeting methylation pathways, oxidative stress, or lipid handling that may hold promise in mitigating liver disease associated with BHMT deficiency.

This research did not include a time-course study in mice younger than 6 months old to determine the onset age of specific liver changes in LKO mice. Further, no experiments were conducted to rescue or reverse the observed liver changes in LKO mice. This study also lacks direct verification of epigenetic modifications, which may contribute to the observed liver changes. Additionally, although we have shown increased hepatic CD68 expression, we did not perform colocalization studies using established macrophage markers such as F4/80 or IBA1 in combination with CD68 to confirm the cellular source of the signal, nor did we identify the phenotype of the inflammatory cells by quantitative methods. We recognize these as limitations of the current study.

## 5. Conclusions

The current study demonstrates that liver-specific deletion of BHMT disrupts hepatic one-carbon metabolism, causing the spontaneous development of hepatic steatosis and liver dysfunction. These findings underscore the essential role of BHMT in maintaining hepatic metabolic homeostasis and reveal a mechanistic link between BHMT deficiency and liver disease progression.

## Data Availability

The original contributions presented in this study are included in the article/Supplementary Materials. Further enquiries can be directed to the corresponding author.

## Supplementary Materials

The following supporting information can be downloaded at https://www.mdpi.com/article/10.3390/biom16040606/s1: Figure S1: Western blot analysis of BHMT expression in kidney lysates of BHMT floxed and LKO mice validating the BHMT LKO mouse model. Figure S2: Body weight, adipose tissue weight, liver weight, and liver-to-body weight ratio in BHMT floxed and LKO mice. Figure S3. Serum triglyceride and non-esterified fatty acid (NEFA) levels. Table S1. List of TaqMan^®^ FAM-labeled primers used for quantitative PCR. Table S2. List of antibodies utilized in the study. Supplementary File S1: The original images of the Western Blots.

## Abbreviations

4-HNE	4-hydroxynonenal
AAALAC	American Association for Accreditation of Laboratory Animal Care
BHMT	betaine-homocysteine methyltransferase
BHMT LKO	Liver-specific BHMT knockout
ALD	alcohol-associated liver disease
CD36	cluster of differentiation 36
CD68	cluster of differentiation 68
CIDEC	cell death-inducing DFFA-like effector C
DCF	dichlorofluorescein
DCFH-DA	2′7′-dichlorodihydrofluorescein diacetate
H&E	hematoxylin and eosin
IL-1β	interleukin 1β
IACUC	Institutional Animal Care and Use Committee
LAL	lysosomal acid lipase
MASLD	metabolic dysfunction-associated liver disease
MDA	malondialdehyde
NEFA	nonesterified fatty acid
SAH	S-adenosylhomocysteine
SAM	S-adenosylmethionine
α-SMA	α-smooth muscle actin
TBARS	thiobarbituric acid-reactive substances
UNMC-TSF	University of Nebraska Medical Center—Tissue Sciences Facility
