# Supplementary material for: Hepatocyte-Specific Deletion of Betaine-Homocysteine Methyltransferase Disrupts Methionine Metabolism and Promotes the Spontaneous Development of Hepatic Steatosis
Source: Biomolecules. 2026 Apr 20;16(4):606. doi: 10.3390/biom16040606 (PMC13113975; doi:10.3390/biom16040606)
Supplement: Supplementary file 1 [file biomolecules-16-00606-s001.zip › Supplementary Figure S1,S2,S3 Table S1,S2.pdf]

Supplementary information

Hepatocyte-specific deletion of betaine-homocysteine methyltransferase disrupts methionine metabolism and promotes the spontaneous development of hepatic steatosis

Ramachandran Rajamanickam [1,2,†], Sathish Kumar Perumal[1,2,†], Ramesh Bellamkonda[1,3], Sundararajan Mahalingam[1,3], Kurt W. Fisher[4], Rolen Quadros[5], Gurumurthy B. Channabasavaiah[5,6], Madan Kumar Arumugam [1,2,7], Karuna Rasineni[1,3], Kusum K Kharbanda[1,2,3,#]

[1]Research Service, Veterans Affairs Nebraska-Western Iowa Health Care System, Omaha, Nebraska, 68105, USA

[2]Department of Internal Medicine, University of Nebraska Medical Center, Omaha, Nebraska, 68198, USA

[3]Department of Biochemistry & Molecular Biology, University of Nebraska Medical Center, Omaha, Nebraska, 68198, USA

[4]Department of Pathology, Microbiology and Immunology, University of Nebraska Medical Center, Omaha, Nebraska, 68198, USA

[5]Mouse Genome Engineering Core Facility, University of Nebraska Medical Center, Omaha, Nebraska, 68198, USA

[6]Current Affiliation: Department of Cell and Molecular Biology, University of Mississippi Medical Center, Jackson, Mississippi, 39216, USA

[7]Cancer Biology lab, Centre for Molecular and Nanomedical Sciences, Sathyabama Institute of Science and Technology, Chennai, 600119, Tamil Nadu, India.

[†] These authors contributed equally to this work

# Correspondence: Kusum K. Kharbanda, Ph.D., Veterans Affairs Nebraska-Western Iowa Health Care System, Research Service (151), 4101 Woolworth Avenue, Omaha, Nebraska, 68105-1850, USA. Tel.: +1-402-995-3752; Fax: +1-402-995-4600. E-mail: **kkharbanda@unmc.edu**

Running title: Liver BHMT deficiency and hepatic dysfunction

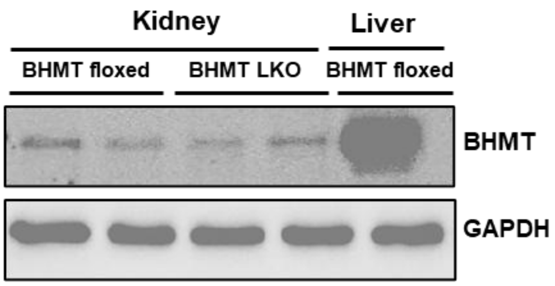

Figure S1. Western blot analysis of BHMT expression in kidney lysates of BHMT floxed and LKO mice validating the BHMT LKO mouse model**.** Liver lysate of a BHMT floxed mouse served as positive control.

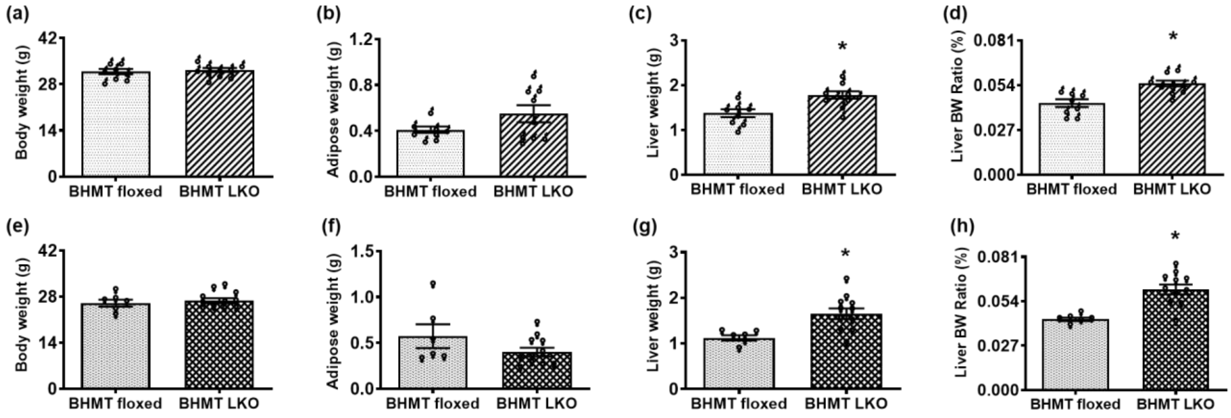

Figure S2. Body weight, adipose tissue weight, liver weight and liver to body weight ratio of 6-month-old chow-fed floxed and liver-specific betaine-homocysteine methyltransferase (BHMT) knockout (LKO) mice. (a,e) Body weight, (b,f) adipose weight, (c,g) liver weight, (d,h) liver to body weight ratio of (a,b,c,d) male and (e,f,g,h) female BHMT floxed and LKO mice. Data are presented as the mean ± SEM (n=6); *$p < 0.05$ versus floxed mice.

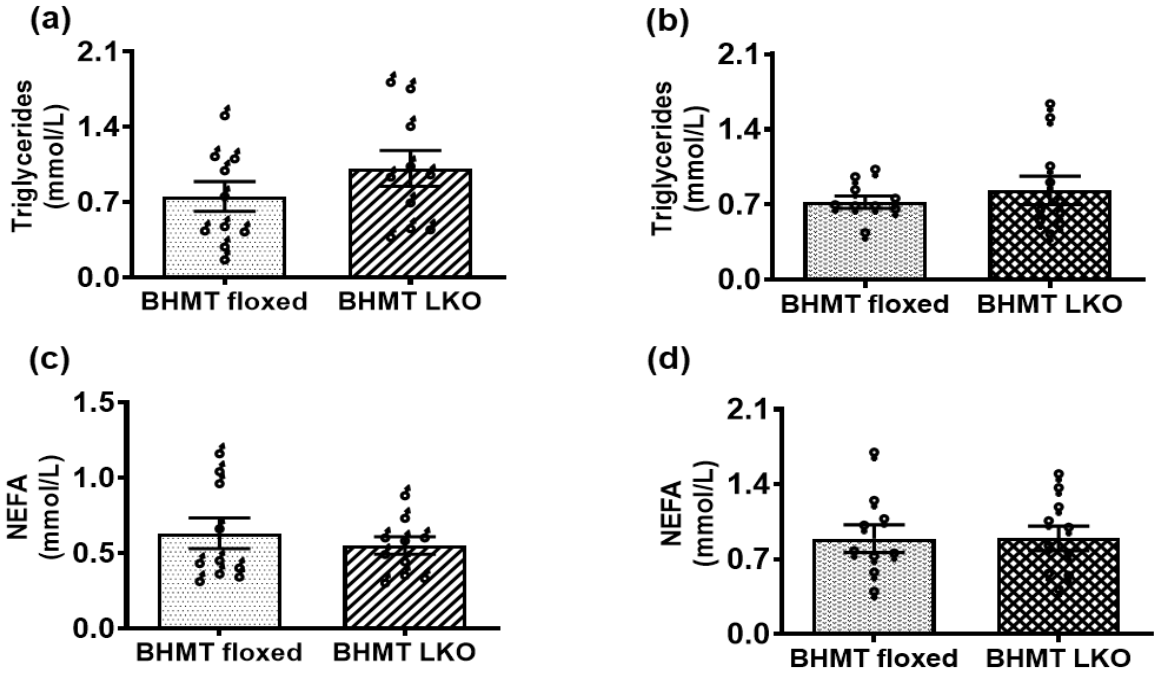

Figure S3. Serum triglycerides, and nonesterified fatty acids (NEFA) levels. (a,b) Serum triglycerides and (c,d) nonesterified fatty acid levels (mmol/Liter) in (a,c) male and (b,d) female BHMT floxed and LKO mice. Data are presented as the mean ± SEM (n=6).

Supplemental Table S1. List of TaqMan® FAM-labeled primers used for quantitative PCR

Gene Name	Assay ID	Manufacturer
Betaine-homocysteine S-methyltransferase (BHMT)	Mm004210521_g1	Applied Biosystems
Cluster of Differentiation 36 (CD36)	Mm00432403_m1	Applied Biosystems
Cell Death-Inducing DFFA-like Effector C (CIDEC)	Mm00617672_m1	Applied Biosystems

Supplemental Table S2. List of antibodies utilized in the study

Primary Antibody	Host	Cat. No.	Manufacturer
β-actin	Mouse	66009-1-Ig	Proteintech
Betaine-homocysteine methyltransferase (BHMT)	Rabbit	Ab96415	Abcam
Cluster of Differentiation 36 (CD36)	Rabbit	100011	Cayman
Cluster of Differentiation 68 (CD68)	Rabbit	76437S	Cell signaling Technology
Collagen II	Mouse	NB600-488	Novus Biologicals
4-Hydroxynonenal (4-HNE)	Mouse	MA5-27570	Invitrogen
Interleukin 1β (IL-1β)	Rabbit	16806-1-AP	Proteintech
Malondialdehyde (MDA)	Mouse	MA5-27560	Invitrogen
Methionine synthase	Rabbit	Ab66039	Abcam
Smooth muscle actin-α (α-SMA)	Mouse	A2547	Sigma

Secondary Antibody	Host	Cat. No.	Manufacturer
Peroxidase AffiniPure Goat Anti-Mouse IgG	Goat	115-035-166	Jackson ImmunoResearch laboratories
Peroxidase AffiniPure Goat Anti-Rabbit IgG	Goat	111-035-144	Jackson ImmunoResearch laboratories
