# Supplementary material for: Hepatocyte-Specific Deletion of Betaine-Homocysteine Methyltransferase Disrupts Methionine Metabolism and Promotes the Spontaneous Development of Hepatic Steatosis
Source: Biomolecules. 2026 Apr 20;16(4):606. doi: 10.3390/biom16040606 (PMC13113975; doi:10.3390/biom16040606)
Supplement: Supplementary file 1 [file biomolecules-16-00606-s001.zip › Supplementary File S1-Supporting Western blots.pdf]

ORIGINAL WESTERN BLOT IMAGES

Figure 2

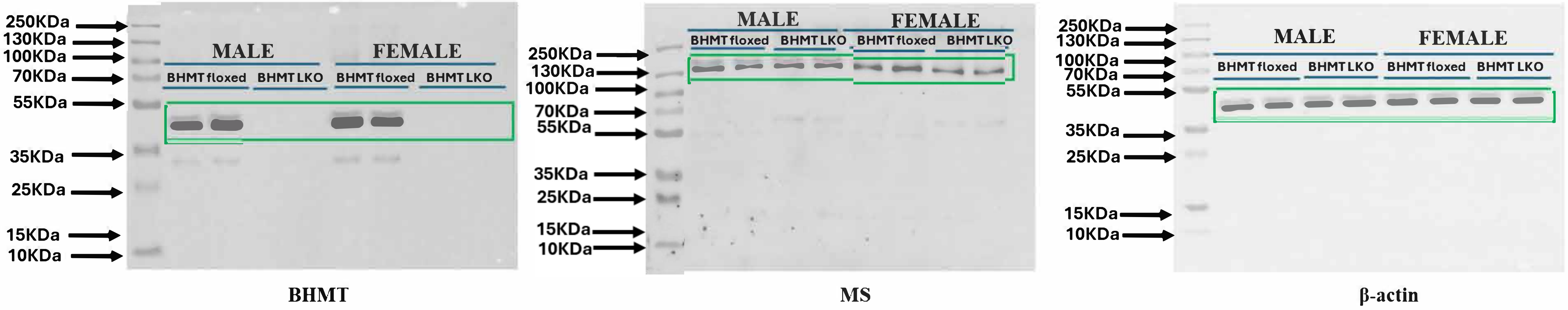

Protein Ladder: Thermo Scientific™ PageRuler™ Plus Prestained Protein Ladder, 10 to 250 kDa (#26619)

Figure 4

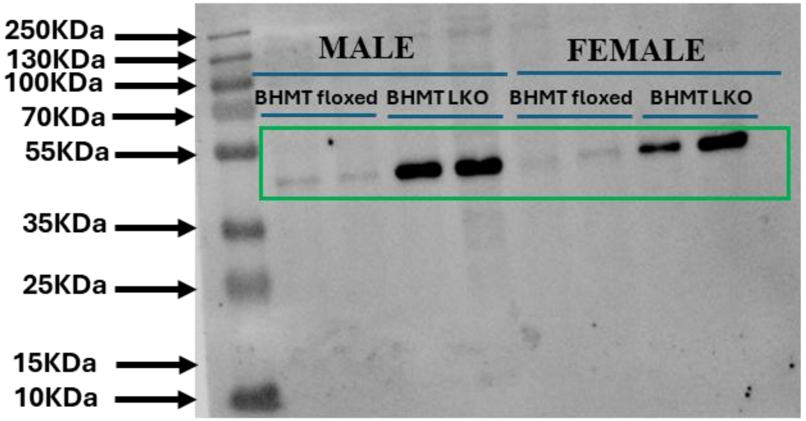

CD36

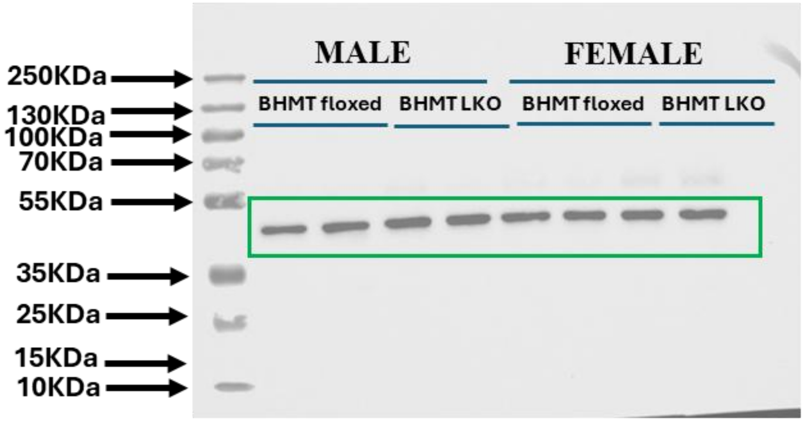

β-actin

Protein Ladder: Thermo Scientific™ PageRuler™ Plus Prestained Protein Ladder, 10 to 250 kDa (#26619)

The same β-actin blot was used for normalizing α-SMA, Collagen II, CD36 and IL-1β, as all the respective Western blots were run using the same lysate and at the same time.

Figure 6

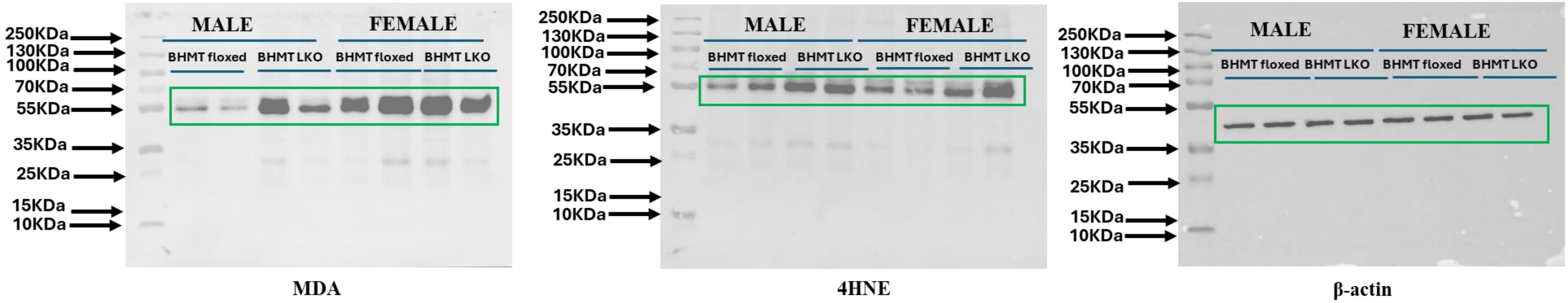

Protein Ladder: Thermo Scientific™ PageRuler™ Plus Prestained Protein Ladder, 10 to 250 kDa (#26619)

Figure 7

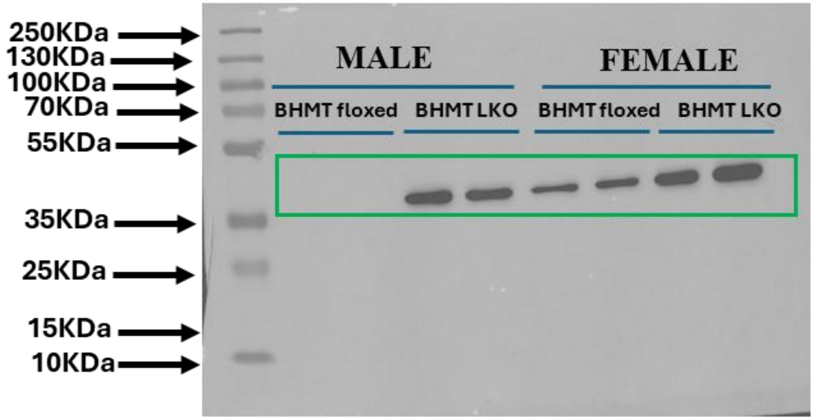

IL-1β

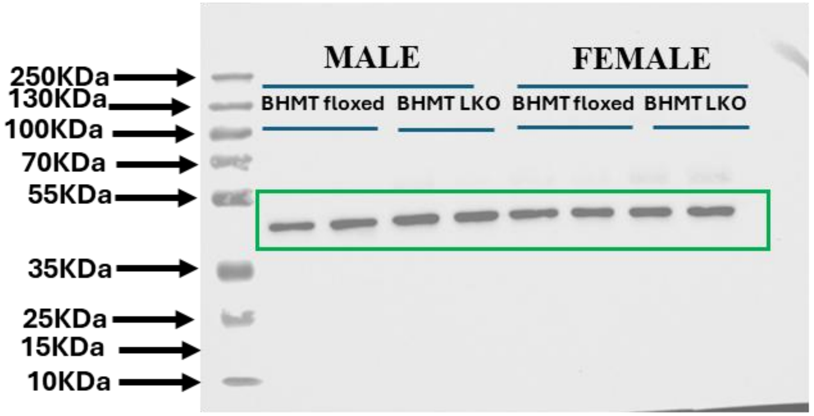

β-actin

Protein Ladder: Thermo Scientific™ PageRuler™ Plus Prestained Protein Ladder, 10 to 250 kDa (#26619)

The same β-actin blot was used for normalizing α-SMA, Collagen II, CD36 and IL-1β, as all the respective Western blots were run using the same lysate and at the same time.

Figure 8

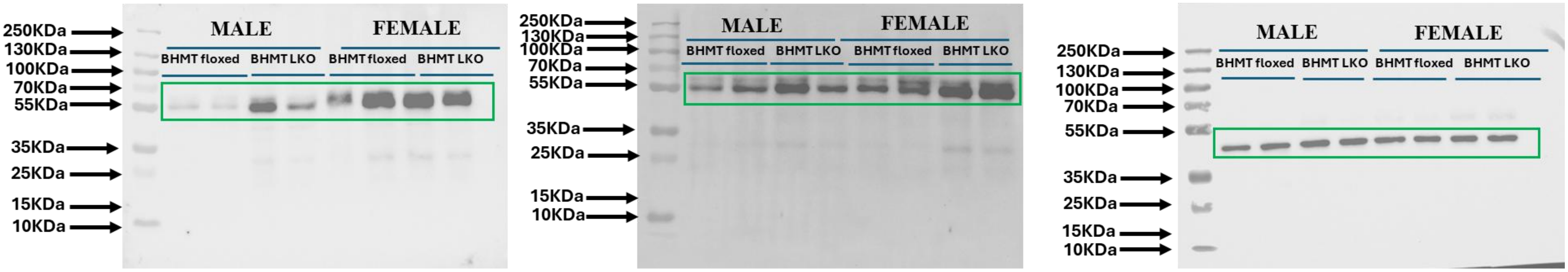

α-SMA   Collagen II   β-actin

Protein Ladder: Thermo Scientific™ PageRuler™ Plus Prestained Protein Ladder, 10 to 250 kDa (#26619)

The same β-actin blot was used for normalizing α-SMA, Collagen II, CD36 and IL-1β, as all the respective Western blots were run using the same lysate and at the same time.

Figure S1

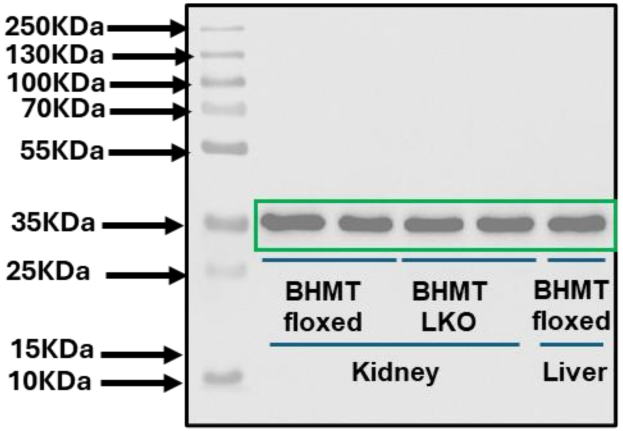

GAPDH – 37kDa

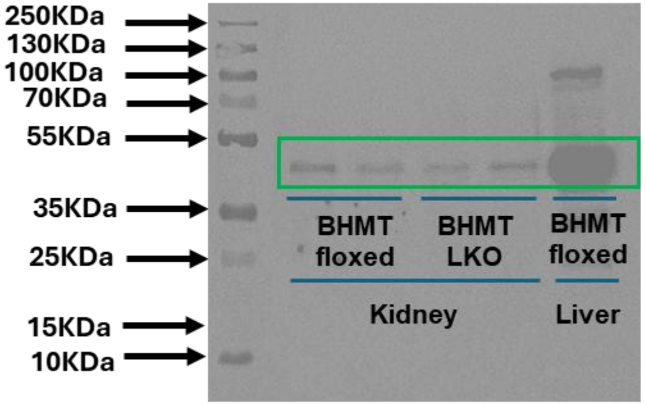

BHMT – 42kDa

Protein Ladder: Thermo Scientific™ PageRuler™ Plus Prestained Protein Ladder, 10 to 250 kDa (#26619)
